# Reactivity and Stability of Metalloporphyrin Complex Formation: DFT and Experimental Study

**DOI:** 10.3390/molecules25184221

**Published:** 2020-09-15

**Authors:** Rimadani Pratiwi, Slamet Ibrahim, Daryono H. Tjahjono

**Affiliations:** 1 Department of Pharmaceutical Analysis and Medicinal Chemistry, Faculty of Pharmacy, Universitas Padjadjaran, Jalan Raya Bandung-Sumedang KM 21, Jatinangor 45363, Indonesia; 2 School of Pharmacy, Bandung Institute of Technology, Jalan Ganesha 10, Bandung 40132, Indonesia; sibrahim@fa.itb.ac.id

**Keywords:** porphyrin, metal, DFT study, global chemical reactivity descriptor, binding constant

## Abstract

The interaction of three cationic porphyrins—*meso*-tetrakis (*N*-methylpyridinium-4-yl) porphyrin (TMPyP), *meso*-tetrakis (1,3-dimethylimidazolium-2-yl) porphyrin (TDMImP), and *meso*-tetrakis (1,2-dimethylpyrazolium-4-yl) porphyrin (TDMPzP)—with five heavy metals was studied computationally, and binding constants were calculated based on data obtained by an experimental method and compared. The reactivity and stability of their complexes formed with lead, cadmium, mercury, tin, and arsenic ions were observed in DFT global chemical reactivity descriptors: the electronic chemical potential (µ), chemical hardness (η), and electrophilicity (ω). The results show that M-TDMPzP has higher chemical hardness and lower electrophilicity compared to M-TMPyP and M-TDMImP, indicating the reaction of TDMPzP with metals will form a more stable complex. Specifically, Cd-TDMPzP complexes can stabilize the system, with a lower energy and electronic chemical potential, higher chemical hardness, smaller electrophilicity, and higher binding constant value compared to Pb-TDMPzP and Hg-TDMPzP. This result suggests that the interaction of the Cd^2+^ ion with TDMPzP will produce a stable complex.

## 1. Introduction

Metallo-complex formation is an important process from an analytical point of view. Porphyrin is a macrocyclic compound that has been widely used in many areas of chemistry. The porphyrin derivatives have become increasingly important reagent candidates in analytical chemistry because of their high sensitivity for determining trace metal elements [1] and their UV-visible spectra with two distinct regions: the near UV and the visible regions [2]. Furthermore, since porphyrin has a large molar absorption coefficient and high stability, it can be applied to separate various kinds of metal ions [3].

Many porphyrin derivatives are synthesized with different substituents and characteristics. A cationic porphyrin is preferable because it is more water soluble and easily handled than water-insoluble reagents. TMPyP (*meso*-tetrakis (*N*-methylpyridinium-4-yl) porphyrin) is one of the cationic porphyrin derivatives that is commercially available, has become a sensitive spectrophotometric reagent for the determination of cadmium [4], and can be used to enhance the absorptive removal of cadmium ions in aqueous solution [5]. TDMImP and TDMPzP are new five-membered-ring cationic porphyrins that are known to be able to bind to DNA [6]. The interaction of these porphyrins as a reagent for metal analysis has recently been reported [7].

The porphyrin core is a tetradentate ligand that has space to coordinate with various kinds of metal ions. The metal ions can fit into the porphyrin core, forming planar metalloporphyrins, or just sit on top of the molecule, forming sitting-atop metalloporphyrins. When the metal ions have ionic radii in the range 55–80 pm, it will take a regular or planar form. On the other hand, if the metal ions are large, with ionic radii over 80–90 pm, they will be out of the ligand plane, forming sitting-atop metalloporphyrins [2]. Metalloporphyrins have many roles in a variety of fields including chemical, medical, biologic, and physic. The Interaction of porphyrin and ligand or metalloporphyrin with other compounds can be observed by Density Functional Theory (DFT) calculation approach [8,9].

Lead (Pb), cadmium (Cd), mercury (Hg), tin (Sn), and arsenic (As) are some examples of heavy metals that have roles as contaminants and are of considerable concern because of their effects on human health [10]. The study of the interaction between porphyrin and heavy metals was performed to predict the stability and reactivity of metalloporphyrin complexes.

**Figure 1:**
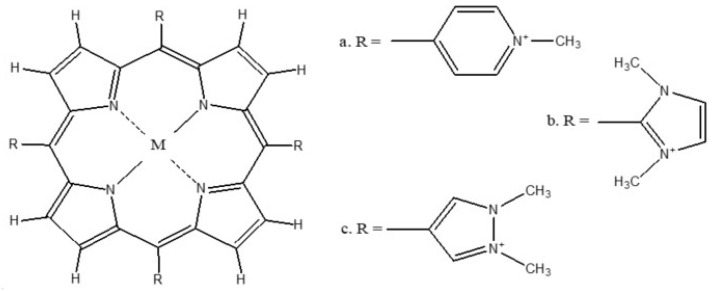
Structure of the cationic porphyrins (a) H_2_TMPyP, (b) H_2_TDMImP, and (c) H_2_TDMPzP, with M = H_2_ for free-base porphyrin and Pb^2+^, Cd^2+^, Hg^2+^, Sn^4+^, As^3+^, and As^5+^ for the complexes.

In the present research, the interactions of three *meso*-substituted cationic porphyrins—*meso*-tetrakis (*N*-methylpyridinium-4-yl) porphyrin (H_2_TMPyP), *meso*-tetrakis (1,3-dimethylimidazolium-2-yl)porphyrin (H_2_TDMImP), and *meso*-tetrakis (1,2-dimethylpyrazolium-4-yl) porphyrin (H_2_TDMPzP)—with five heavy metals (Figure 1) has been analyzed computationally by the DFT (Density Functional Theory) method based on their electronic and structural properties. The parameters that were observed were the chemical hardness (η), electronic chemical potential (µ), and electrophilicity (ω), which is also known as the DFT global chemical reactivity [11]. In addition, the bond length (M-NPh and M-PPh) and the angle between a metal and a porphyrin plane (M-plane), as a structural parameter, were also calculated. Furthermore, the binding constants of the complexes of Pb-TDMPzP, Cd-TDMPzP, and Hg-TDMPzP were determined experimentally. The results showed that the interaction of TDMPzP with metals formed more stable complexes and TDMPzP-Cd had a higher binding constant compared to TDMPzP-Pb and TDMPzP-Hg.

## 2. Results and Discussion

### 2.1. Calculated Structure of Metalloporphyrin Complex

All of the molecules were optimized to obtain the most stable conformations. With the lowest energy state, good stability of molecules is achieved. Some of the molecules or atoms with high energy levels are mutually joined by releasing their energy to become more stable. Porphyrin and metal ions will collide to react. If the collision produces enough energy to exceed the activation energy, porphyrin−metal complexes will be produced at a lower energy level than that for free base porphyrin.

As shown in Table 1, all the porphyrin−metal complexes have lower energies than the free base form. It is predicted that the reaction between cationic porphyrin and a metal ion can take place spontaneously. In general, TMPyP has higher energy, not only in its free base form but also in its complex form, compared to the energies of TDMImP and TDMPzP. TDMImP and TDMPzP have similar energies in both forms. This shows that the optimum geometries of TDMImP and TDMPzP have better molecule stability than TMPyP. In other words, both TDMImP and TDMPzP have better molecule stability than TMPyP.

The length of the M-NPh and M-PPh bonds and the angle between a metal and a porphyrin plane (M-plane) were calculated to predict the stability of the metalloporphyrin complexes. The stability of metalloporphyrin complexes is affected by the ionic radii of the metals. The ionic radii of the heavy metals are 118, 95, 102, 69, 58, and 46 pm for Pb^2+^, Cd^2+^, Hg^2+^, Sn^4+^, As^3+^, and As^5+^, respectively [12]. Based on their radii, it follows that Pb^2+^, Cd^2+^, and Hg^2+^ will form sitting-atop metalloporphyrins because their ionic radii are over 80–90 pm, while Sn^4+^, As^3+^, and As^5+^ will form regular metalloporphyrins.

**Figure 2:**
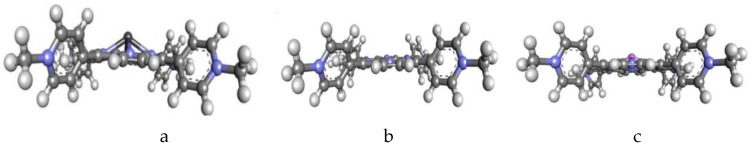
Visualization of the optimized structure of TMPyP complexes with the metal ions (**a**) Pb^2+^, (**b**) Cd^2+^, and (**c**) As^3+^, where (**a**,**c**) are sitting-atop metalloporphyrins and (**b**) is a regular metalloporphyrin.

The results show that Cd^2+^ and Sn^4+^ can fit into all of the cationic porphyrin core and form regular metalloporphyrins (Figure 2b). This is due to the ionic radii of these metals, which have sufficient sizes to fit into the inner cavity of porphyrin; thus, the distance from the M-plane is 0 (Table 2). Both Pb^2+^ and Hg^2+^ have large ionic radii, and because the porphyrin core does not have enough space to accommodate these metals, both metals form sitting-atop metalloporphyrins, with the distances from the M-plane being almost similar for both metals. Both As^3+^ and As^5+^ have the smallest ionic radii of the studied metals, but they tend to move out from the porphyrin plane (Figure 2c), and the structural parameters for the As^5+^ ion in the TDMPzP core cannot be determined. This may be due to the valence electrons of the As metal ion not being appropriate for interaction with the nitrogen of the porphyrin core.

The calculation of the DFT global chemical reactivity descriptor is important in conceptual DFT, as these values can be used to understand the relationships between the structure, stability, and reactivity of a molecule [13]. These parameters are obtained based on the calculation of the HOMO (Highest Occupied Molecular Orbital) and LUMO (Lowest Unoccupied Molecular Orbital) energy of the optimized molecule.

**Figure 3:**
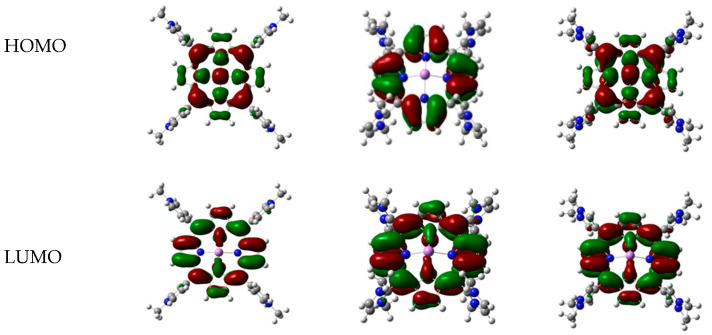
Electron distribution of As^3+^ with TMPyP (**left**), TDMImP (**center**), and TDMPzP (**right**).

The HOMO and LUMO contour surfaces of metalloporphyrin complexes show the electron distributions between porphyrin and a metal ion. As shown in Figure 3, the electron distribution of metalloporphyrin complexes occurs around the porphyrin core. This means that interaction between a metal ion and porphyrin takes place in the porphyrin core. In general, the main species of the HOMO of a metalloporphyrin complex come from the d orbital of the metal ion, while the main species of the LUMO come from the p orbital of the porphyrin. This shows that there is a charge transfer from the metal to porphyrin.

Chemical hardness (η) is defined as the molecular resistance needed to change or deform the number of electrons, and it is correlated with the stability and reactivity of a chemical system [14,15]. This parameter is calculated as in Equation (1). The hardness is the energy gap between the two frontier orbitals, HOMO and LUMO [16]. A hard molecule has a large HOMO−LUMO gap. With a large gap, a molecule will be more stable and also need much excitation energy to reach the manifolds of excited states [17]. As shown in Table 3, M-TDMPzP has a high chemical hardness compared to the other compounds. Thus, M-TDMPzP is a hard molecule and less reactive than the others.

The electronic chemical potential (µ) measures the tendency of electrons to escape from an equilibrium system [18]. This parameter is described as the electronegativity of the molecule [19]. Based on a calculation using Equation (2), M-TDMImP has a lower electronic chemical potential value than the others. This shows that this molecule is more stable because it will retain the electron to escape from the system.

Electrophilicity (ω) describes the capability of species to accept electrons [20]. It measures the change in energy of an electrophile when it comes in contact with a nucleophile [21]. This parameter is calculated by combining chemical hardness and electronic chemical potential (Equation (3)). The electrophilicity tends to decrease in the order M-TDMImP, M-TMPyP, and then M-TDMPzP. This shows that M-TDMPzP is more stable and less reactive in terms of accepting an electron.

Based on the DFT global chemical reactivity descriptor, M-TDMPzP has the highest chemical hardness and lowest electrophilicity. It does not have the lowest energy value, but the energies of M-TDMPzP and M-TDMImP are similar. M-TDMPzP has a large HOMO−LUMO gap, which indicates that this complex will be more stable. All of these results suggest that the interaction of TDMPzP with metals will form complexes more stable compared to those formed with the others.

Based on the calculation of the DFT global chemical reactivity descriptors, the interaction of TDMPzP with metal will form complexes more stable compared to those formed with the others. Furthermore, TDMPzP was synthesized to analyze the binding constant of a complex of Pb-TDMPzP, Cd-TDMPzP, and Hg-TDMPzP.

### 2.2. Calculation of Binding Constants

TDMPzP was synthesized to analyze the binding constant of a complex of Pb-TDMPzP, Cd-TDMPzP, and Hg-TDMPzP. If we observe only the structural parameters of complex Pb-TDMPzP, Cd-TDMPzP, and Hg-TDMPzP as shown in Table 2, the Cd^2+^ ion can fit into the porphyrin core, forming regular metalloporphyrins, while the Pb^2+^ and Hg^2+^ ions are located out of the ligand plane, forming sitting-atop metalloporphyrins. In addition, Cd-TDMPzP has lower energy compared to Pb-TDMPzP and Cd-TDMPzP (Table 4). The global chemical reactivity descriptor shows that Cd-TDMPzP had the smallest energy and electronic chemical potential (µ) value, larger chemical hardness (η), and the smallest electrophilicity (ω). It is predicted that the Cd-TDMPzP complex is more stable and less reactive in terms of accepting an electron. This result correlates with the binding constant values shown in Table 4. Cd-TDMPzP has the highest binding constant of 5.07 × 10^7^ M^−1^, implying that TDMPzP will form a more stable metalloporphyrin complex when reacted with Cd^2+^.

## 3. Experimental Section

### 3.1. Computational Methods

The heavy metals that were used as a model in this study were Pb^2+^, Cd^2+^, Hg^2+^, Sn^4+^, As^3+^, and As^5+^. These heavy metals with different valences were selected due to their common role as contaminants in food and water. All of the molecules were optimized using Gaussian 09 and GausView 05. The calculations were performed using the DFT method with the B3LYP level and 6−31G basis set for free-base porphyrin and the LANL2DZ basis set for the metalloporphyrin complexes. After the molecules were optimized, the calculation of the structural parameter was performed to predict the stability of the metalloporphyrin complexes. The HOMO and LUMO energies were then calculated to determine the DFT global chemical reactivity descriptor.

DFT global chemical reactivity descriptors, including the chemical potential (µ), global hardness (η), and electrophilicity (ω), are calculated using the following equations [16]:

η = (I − A)/2(1)

µ = (I + A)/2(2)

ω = µ^2^/2 η (3)

where I is the ionization energy and A is the electron affinity of the electron system (neutral or charged) studied. These parameters are calculated using the orbital theory approach by measuring the LUMO and HOMO energies for the ionization energy and the electron affinity, respectively [16].

### 3.2. Calculation of Binding Constant

TDMPzP sulfonate was synthesized with a slight modification of a reported literature procedure by Daryono et al., Romera et al., and Pasternack et al., using the Adler method [6,22,23]. The binding constant of the complex of Pb-TDMPzP, Cd-TDMPzP, and Hg-TDMPzP was determined using the spectrophotometric titration method and estimated by using the nonlinear curve as the following expression [24]:

(4) $$Y=Y_{0}\;+\;\frac{Y_{lim}\;-\;Y_{0}\;}{2}\left\{1\;+\;\frac{C_{M}}{C_{L}}\;+\;\frac{1}{K_{S}C_{L}}\;-\;[(1\;+\;\frac{C_{M}}{C_{L}}\;+\;{\frac{1}{K_{S}C_{L}}\;)}^{2}-\;4\;{\frac{C_{M}}{C_{L}}\;]}^{1/2}\right\}$$

Here, *Y* refers to the absorbance of a ligand in the presence of a metal; *Y*_0_ represents the absorbance of the free ligand; *C_M_* and *C_L_* are the concentrations of the metal and ligand, respectively; and *Ks* is the binding constant.

## 4. Conclusions

The study of the interaction of molecules based on the calculated DFT global chemical reactivity descriptors help in predicting the stability and reactivity of metalloporphyrin complex formation. The ionic radii of the metal ions influence the stability of the metalloporphyrin complexes. Large metal ions will form sitting-atop metalloporphyrins, which are kinetically labile compared to regular metalloporphyrins. Based on the calculation of the DFT global chemical reactivity descriptors, the interaction of TDMPzP with metals will form a more stable complex. Furthermore, the experimental results validate our calculation that TDMPzP would form a more stable metalloporphyrin complex when it reacted with a Cd^2+^ ion, with a binding constant of 5.1 × 10^7^ M^−1^.

## Figures and Tables

**Table 1:** Molecular energies of the optimized structures of cationic porphyrin complexes.

No.	TMPyP (Energy, Hartree)		TDMImP (Energy, Hartree)		TDMPzP (Energy, Hartree)	
**1.**	Base	−2135.8	Base	−2204.7	Base	−2204.6
**2.**	Pb-TMPyP	−2138.3	Pb-TDMImP	−2207.3	Pb-TDMPzP	−2207.2
**3.**	Cd-TMPyP	−2182.9	Cd-TDMImP	−2251.9	Cd-TDMPzP	−2251.8
**4.**	Hg-TMPyP	−2177.5	Hg-TDMImP	−246.5	Hg-TDMPzP	−2246.4
**5.**	Sn-TMPyP	−2137.1	Sn-TDMImP	−2205.9	Sn-TDMPzP	−2206.0
**6.**	As^3+^-TMPyP	−2140.4	As^3+^-TDMImP	−2209.3	As^3+^-TDMPzP	−2209.3
**7.**	As^5+^-TMPyP	−2138.9	As^5+^-TDMImP	−2207.8	As^5+^-TDMPzP	−2207.9
**Average**	M-TMPyP	−2152.5	M-TDMImP	−2221.5	M-TDMPzP	−2221.4

**Table 2:** Calculated structural parameters (bond lengths and angles) of three cationic porphyrin complexes.

	Parameter	Pb^2+^	Cd^2+^	Hg^2+^	Sn^4+^	As^3+^	As^5+^
**TMPyP**	**M-Nph (Å)**	2.370	2.163	2.244	2.046	2.128	2.107
	**M-Plane (Å)**	1.215	0	0.540	0	0.648	0.686
	**Bond Angle (M-Plane)**	30.84°	0	13.92°	0	17.73°	19.00°
**TDMImP**	**M-Nph (Å)**	2.370	2.159	2.247	2.048	2.128	2.136
	**M-Plane (Å)**	1.108	0	0.576	0	0.652	0.660
	**Bond Angle (M-Plane)**	27.87°	0	14.85°	0	17.84°	18.00°
**TDMPzP**	**M-Nph (Å)**	2.366	2.158	2.243	2.045	2.127	−
	**M-Plane (Å)**	1.101	0	0.544	0	0.651	−
	**Bond Angle (M-Plane)**	27.73°	0	14.04°	0	17.82	−

M-NPh: the average bond length of the metal−nitrogen porphyrin. M-Plane: the length of the distance between the metal and porphyrin plane. ∠M-Plane: the angle between the metal and porphyrin plane.

**Table 3:** Global chemical reactivity descriptor for cationic porphyrin−metal complexes (units in au).

	Parameter	HOMO	LUMO	µ	η	ω
**TMPyP**	P	−0.509	−0.414	−0.462	0.048	2.223
	P-Pb	−0.504	−0.417	−0.461	0.044	2.415
	P-Cd	−0.513	−0.418	−0.466	0.048	2.262
	P-Hg	−0.512	−0.418	−0.465	0.047	2.300
	P-Sn	−0.747	−0.638	−0.693	0.055	4.366
	P-As^3+^	−0.626	−0.523	−0.575	0.052	3.179
	P-As^5+^	−0.862	−0.813	−0.838	0.025	14.045
	Average	−0.627	−0.538	−0.583	0.045	4.761
**TDMImP**	I	−0.555	−0.450	−0.503	0.053	2.387
	I-Pb	−0.551	−0.452	−0.502	0.050	2.520
	I-Cd	−0.557	−0.452	−0.505	0.053	2.406
	I-Hg	−0.556	−0.452	−0.504	0.052	2.442
	I-Sn	−0.768	−0.688	−0.728	0.040	6.625
	I-As3	−0.676	−0.572	−0.624	0.052	3.744
	I-As5	−0.866	−0.830	−0.848	0.018	19.975
	Average	−0.662	−0.574	−0.618	0.044	6.285
**TDMPzP**	Pz	−0.496	−0.391	−0.444	0.053	1.860
	Pz-Pb	−0.490	−0.396	−0.443	0.047	2.088
	Pz-Cd	−0.499	−0.395	−0.447	0.052	1.921
	Pz-Hg	−0.496	−0.396	−0.446	0.050	1.989
	Pz-Sn	−0.740	−0.632	−0.686	0.054	4.357
	Pz-As3	−0.619	−0.515	−0.567	0.052	3.091
	Pz-As5	−0.830	−0.780	−0.805	0.025	12.961
	Average	−0.612	−0.519	−0.566	0.046	4.401

**Table 4:** Binding constants of M-TDMPzP complexes.

Complexes	Binding Constant (M^−1^)
Pb-TDMPzP	3.0 × 10^7^
Hg-TDMPzP	3.3 × 10^7^
Cd-TDMPzP	5.1 × 10^7^

## References

[1] Li Z., Pan J. (2020). Advances in synthesis and application of the derivatives of porphyrin as a reagent in analytical chemistry. Rev. Anal. Chem..

[2] Giovannetti R., Uddin J. (2012). The use of spectrophotometry UV-VIS for the study of porphyrins. Macro to Nano Spectroscopy.

[3] Ishi H., Katsuhiko S., Yasuhiro S., Hidemasa K. (1982). Spectrophotometric and analogue derivative spectrophotometric determination of ultramicro amounts of cadmium with cationic porphyrins. Talanta.

[4] Hu Q., Yang G., Li H., Tai X. (2004). Study on determination of seven transition metal ions in water in food by microcolumn high performance liquid chromatography. Bull. Korean Chem. Soc..

[5] Zhang L., Zhao Y.H., Bai R.B. (2011). Development of a multifunctional membrane for chromatic warning and enhanced adsorptive removal of heavy metal ions: Application to cadmium. J. Membr. Sci..

[6] Daryono H.T., Takehiro A., Naoki Y., Hidenari I. (1999). Cationic porphyrins bearing diazolium rings: Synthesis and their interaction with calf thymus DNA. Biochim. Biophys. Acta.

[7] Pratiwi R., Nguyen M.P., Ibrahim S., Yoshioka N., Henry C.S., Tjahjono D.H. (2017). A selective distance-based paper analytical device for copper(II) determination using a porphyrin derivative. Talanta.

[8] Gonzalez J.C., Mondal S., Ocayo F., Maturana R.G., Muñoz-Castro A. (2019). Nature of C60 and C70 Fullerene Encapsulation in a Porphyrin-and Metalloporphyrin-Based Cage: Insights from Dispersion Corrected DFT Calculations. Int. J. Quant. Chem..

[9] Ulloa C.O., Ponce-Vargas M., Muñoz-Castro A. (2018). Formation of Coinage-Metal Fullerene Adducts. Evaluation of the Interaction Nature between Triangular Coinage Metal Complexes (M3 = Cu, Ag, and Au) and C60 through Relativistic Density Functional Theory Calculations. J. Phys. Chem. C.

[10] Merian E., Anke M., Inhat M., Stoeppler M., Merian E., Anke M., Inhat M., Stoeppler M. (2004). Metals and their compound. Elements and Their Compounds in the Environment.

[11] Chandrakumar K., Sourav P. (2002). The concept of density functional theory based descriptors and its relation with the reactivity of molecular systems: A semi-quantitative study. Int. J. Mol. Sci..

[12] Smith D.W. (1990). Inorganic Substances: A Prelude to the Study of Descriptive Inorganic Chemistry.

[13] Chattaraj P.K., Vijayaraj R., Subramanian V. (2009). Comparison of global reactivity descriptors calculated using various density functionals: A QSAR perspective. J. Chem. Theory Comput..

[14] Parr R.G., Pearson R.G. (1983). Absolute hardness: Companion parameter to absolute electronegativity. J. Am. Chem. Soc..

[15] Pearson R.G. (2005). Chemical hardness and density functional theory. J. Chem. Sci..

[16] Geerlings P., Proft F.D., Langenaeker W. (2003). Conceptual density functional theory. Chem. Rev..

[17] Pearson R.G. (1997). Chemical Hardness: Density Functional Theory.

[18] Chattaraj P.K., Nath S., Maiti B., Patrick B., Hans D.W., Wilfried L., Jan P.T. (2004). Reactivity Descriptors. Computational Medicinal Chemistry for Drug Discovery.

[19] Mebi C.A. (2011). DFT study on structure, electronic properties, and reactivity of cis-isomers of [(NC_5_H_4_-S)_2_Fe(CO)_2_]. J. Chem. Sci..

[20] Feng X.T., Yu J.G., Lei M., Fang W.H., Liu S.B. (2009). Toward understanding metal-binding specificity of porphyrin: A conceptual density functional theory study. J. Phys. Chem. B.

[21] Chattaraj P.K., Santanab G. (2009). Electrophilicity index within a conceptual DFT framework. Annu. Rep. Prog. Chem. Sect. C Phys. Chem..

[22] Romera C., Sabater L., Garofalo A., Dixon I.M., Pratviel G. (2010). Interaction of cationic nickel and manganese porphyrins with the minor groove of DNA. Inorg. Chem..

[23] Pasternack R.F., Huber P.R., Boyd P., Engasser G., Francesconi L., Gibbs E., Fasella P., Venturo G.C., Hinds L.d. (1971). On the aggregation of meso-substituted water-soluble porphyrins. J. Am. Chem. Soc..

[24] Valeur B., Bernard-Santos M.N. (2001). Molecular Fluorescence: Principles and Application.

